# Perceptions and Experiences of Physiotherapists Treating Trismus in Head and Neck Cancer Patients: Findings from a Spanish Web-Based Survey

**DOI:** 10.3390/jcm14197092

**Published:** 2025-10-08

**Authors:** Ernesto Anarte-Lazo, Carlos Bernal-Utrera

**Affiliations:** 1 Faculty of Health, UNIE University, 28015 Madrid, Spain; 2 Physiotherapy Department, Faculty of Nursing, Physiotherapy and Podiatry, University of Seville, 41009 Seville, Spain; cbutrera@us.es

**Keywords:** head and neck cancer, trismus, physiotherapists, qualitative study

## Abstract

**Background:** Trismus is a frequent and debilitating complication in people with head and neck cancer (HNC), often arising after surgery or radiotherapy. Despite the key role physiotherapists play in rehabilitation, little is known about their perspectives and clinical approaches. This study aimed to explore physiotherapists’ experiences, perceptions, and treatment strategies in the management of treatment-induced trismus in HNC. **Methods:** A qualitative, cross-sectional study was conducted via a web-based self-administered questionnaire, developed in accordance with the CHERRIES guidelines. The survey combined closed- and open-ended questions across five thematic areas: sociodemographic, clinical experience, treatment practices, barriers, and medical devices. An inclusion algorithm ensured participation of physiotherapists with expertise in trismus. Quantitative data were analyzed descriptively; open responses underwent inductive thematic analysis. **Results:** Of 72 invited physiotherapists, 31 responded and 18 met inclusion criteria. Participants reported integrating manual therapy (100%) and therapeutic exercise (100%) into treatment, with more selective use of medical devices (77%). Barriers to implementation included lack of functional tools, dental status limitations, patient adherence issues, and socioeconomic constraints. Many highlighted that current devices often fail to mimic functional mastication, potentially overloading central incisors and limiting functional carryover. **Conclusions:** This qualitative study reveals limited device use, perceived design shortcomings, and the influence of dental status on functional recovery. Findings highlight the need for device innovation, interdisciplinary coordination, and protocols tailored to patient-specific barriers. Future research should explore combined approaches and include patient perspectives.

## 1. Introduction

Head and neck cancer (HNC) includes a group of malignancies that affect the upper aerodigestive tract, primarily the oral cavity, pharynx, and larynx. Globally, HNC represents about 5% of all cancers, with approximately 800,000 new cases diagnosed each year. Estimates suggest that this number will exceed one million annually in the coming years [1]. In Europe, the incidence is around 150,000 new cases per year, with men being affected about twice as often as women [2].

Treatments for HNC, such as radiotherapy, surgery, and chemotherapy, have improved survival rates over the past decades. However, these therapies frequently result in long-term complications that significantly compromise patients’ daily functioning [3,4]. One of the most common and disabling sequelae is trismus, generally defined as a maximal interincisal opening of less than 35 mm [5]. Trismus prevalence is estimated to range between 32% and 44% in patients treated with radiotherapy, with variability depending on tumor location, dose distribution, treatment modality, and the operational definition used across studies [6].

Trismus often results from fibrosis affecting the masticatory muscles, and its consequences go beyond a physical limitation: it can interfere with eating, speaking, maintaining oral hygiene, and even social interaction [7]. As a result, managing trismus effectively is a key component of long-term care in these patients. Rehabilitation, particularly through therapeutic exercise (TE), remains the mainstay of trismus treatment. A recent review highlighted exercise as the most used intervention, although manual therapy (MT) techniques have also been explored [8]. The types of exercises used range from passive stretching and active range of motion to structured training programs involving medical devices. Some randomized controlled trials have compared these approaches [9,10], and a recent systematic review concluded that while exercise can improve mouth opening in patients who already have trismus, it is less effective in preventing it [11].

In recent years, medical devices have become increasingly common in the rehabilitation of trismus. Nonetheless, most are designed to provide passive or active stretching to counteract the fibrotic changes caused by radiotherapy or surgery [12]. Some newer devices go a step further by resisting jaw movements, aiming not only to improve range of motion but also to retrain muscle function [13,14]. However, their design is not adapted to the dental arch, and the resistance is offered in central incisors.

In this context, physiotherapists play a crucial role in managing trismus. They are responsible for assessing functional limitations, designing individualized exercise programs, and guiding patients throughout their recovery process. However, despite their central role, very little is known about how physiotherapists themselves perceive the treatment of trismus in people with HNC. To date, no studies have explored their views, experiences, or clinical reasoning regarding this condition. This represents a gap in the literature, especially considering the growing emphasis on personalized rehabilitation and patient-centered care [15]. Understanding how physiotherapists approach trismus could help identify not only which interventions are used most frequently, but also what challenges they face in real-world clinical settings—such as limited access to devices, uncertainty about optimal dosage, or variability in patient adherence.

Although several randomized controlled trials and systematic reviews have examined exercise-based interventions and device use in trismus rehabilitation [16,17], these studies have primarily focused on clinical outcomes (e.g., changes in mouth opening) rather than on how physiotherapists reason, adapt, and implement such approaches in practice. Moreover, most device-oriented research has been industry-driven, with limited exploration of whether these tools are perceived as functional, feasible, or acceptable in real-world rehabilitation. To date, no studies have investigated the perspectives of physiotherapists—professionals who not only prescribe and adapt therapeutic exercise, but also integrate manual therapy, evaluate barriers such as dental status, and liaise with oncologists and dentists in multidisciplinary teams. This gap restricts our understanding of how evidence-based interventions are actually delivered, what challenges limit their effectiveness, and how innovation in device design and clinical protocols can be informed by frontline expertise. Addressing this gap is therefore critical to optimize patient outcomes and promote interdisciplinary strategies in head and neck cancer rehabilitation.

This study aims to address that gap by exploring the experiences, perceptions, and treatment strategies of physiotherapists working with patients who have developed trismus following cancer treatment. Using a qualitative approach based on online surveys, we sought to gather insights from clinicians directly involved in this field. The objectives of the study were to: (a) identify current physiotherapy practices in the management of treatment-induced trismus in head and neck cancer; (b) to understand physiotherapists’ perceptions of the effectiveness, feasibility, and limitations of these interventions, and how these perceptions shape their clinical decisions.

## 2. Materials and Methods

### 2.1. Study Design

A qualitative survey study was conducted with a cross-sectional design, using an online self-administered questionnaire. The methodology was designed and reported based on the Checklist for Reporting Results of Internet E-Surveys (CHERRIES) translated into Spanish [18]. The aim of this study was to explore perceptions, experiences and clinical practices regarding the management of treatment-induced trismus in people with HNC coming from Spanish physiotherapists’ experts in this field.

### 2.2. Participants and Recruitment

Participants were licensed physiotherapists currently in clinical practice and/or research concerning trismus. We used a combination of convenience and snowball sampling, which facilitated the recruitment of physiotherapists with expertise in temporomandibular disorders TMD and HNC. While we acknowledge that this approach may introduce selection bias and limit generalizability, it was considered appropriate given the scarcity of clinicians with this specialized expertise. This limitation is further discussed in the Section 4. Through the 8–10 min survey, we ensured that they had expertise also in the management of treatment-induced trismus in people with HNC. Participants were invited to participate personally based on: (a) web-based search; (b) publications in books and research papers; (c) prior knowledge from the research team; (d) recommendations coming from invited experts; (e) contacting physiotherapy scientific societies. Participation was voluntary and anonymous. The survey was open from 15 July 2025–9 August 2025. All invitations were performed by the research team by e-mail/private messages by LinkedIn). With the aim of ensuring that all finally included participants were experts, a short algorithm was established and can be found in the Section 3 with the results of the recruitment procedure.

### 2.3. Questionnaire Development

The questionnaire was developed using Google Forms (Google LLC, Mountain View, CA, USA) by a team of physiotherapists with clinical and research experience in oncology rehabilitation. The questionnaire was developed based on previous literature on trismus management and rehabilitation [11,12] and a clinical book in the field [19], complemented by expert consultation with three physiotherapists specializing in TMD and HNC. Items were drafted to cover domains including treatment practices, perceived barriers, facilitators, and device use.

A pilot test was conducted with five physiotherapists not included in the final sample to ensure clarity and content validity. As a result, several questions were rephrased for simplicity, the order of sections was adjusted to improve readability, and an additional open-ended item on device-related experiences was included.

The final questionnaire included both closed- and open-ended questions, organized into five sections that can be found in Table 1. An example of questions for each domain can be found at the end of each column. The full survey can be found in Supplementary Materials File S1.

### 2.4. Ethical Considerations and Informed Consent

Before accessing the questionnaire, participants were presented with an information sheet detailing the purpose of the study, data protection measures, and the voluntary nature of participation. Informed consent was obtained by selecting an acceptance checkbox. Only participants who provided the consent could proceed to the survey. This study adheres to the Declaration of Helsinki.

### 2.5. Data Protection and Duplicate Entry Prevention

To prevent duplicate entries, responses were screened manually for similarities in submission time, sociodemographic data, and professional profiles. IP addresses were not collected, and no personally identifiable information was gathered. All data were stored in a secure institutional account protected by a password.

### 2.6. Data Analysis

Quantitative variables were analyzed using descriptive statistics (means, standard deviations, frequencies and percentages) using SPSS software (Version 31.0.0 for MacOS).

Open-ended responses were analyzed using inductive thematic analysis following the steps proposed previously [20]: familiarization with data, initial coding, searching for themes, reviewing themes, defining and naming themes, and writing the report. Two researchers independently coded the transcripts, and an initial codebook was developed inductively. Codes were iteratively refined through discussion, and disagreements were resolved by consensus. Inter-rater reliability was calculated using Cohen’s kappa (κ = 0.78), indicating substantial/almost perfect agreement. An audit trail documenting coding decisions and theme development was maintained to enhance transparency.

Data saturation was monitored throughout the inductive thematic analysis. After coding the final three responses, no new codes or themes emerged, suggesting that saturation had been reached. This supported the adequacy of the sample size (*n* = 18) to capture the diversity of physiotherapists’ experiences.

## 3. Results

A total of 72 professionals were invited to participate in our survey through an email including the Google Forms link. Among them, 31 completed the questionnaire, and following our algorithm, 18 participants were included in our analysis because of their relevant experience in the management of treatment-induced trismus in HNC.

**Figure 1:**
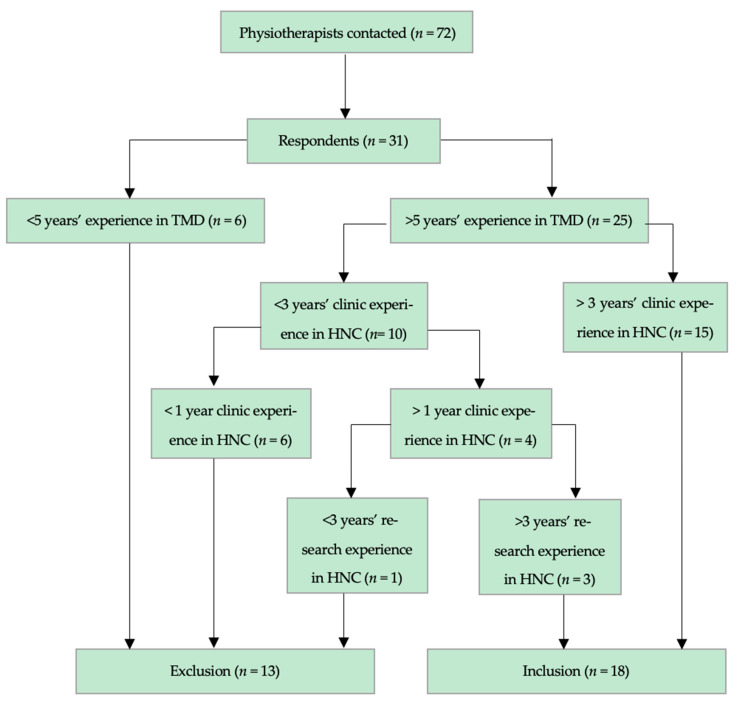
Flowchart of recruitment procedure. HNC: Head & Neck Cancer; TMD: Temporomandibular Disorders.

A flow diagram of included and excluded participants can be found in Figure 1.

### 3.1. Sociodemographic Characteristics and Professional Information

A total of 18 physiotherapists participated in the survey. Participants had a mean age of 44 years (SD: 8.7), and 7 were women (39%). The average number of years of professional experience in patients with TMD was 11 (SD: 3.4) years.

### 3.2. Experience in HNC Management and General Associated Features of Patients

Among included participants, six of them reported to have worked for more than five years specifically with people with HNC; eight reported experience in this field of between three and five year;, and only three reported clinic experience of one to three years but with more than three years in research. Regarding experience in terms of the number of patients with HNC, only three reported having managed fewer than 20 patients, with six reporting more than 50 patients and no one more than 100 patients.

When asked which were the three most common clinical features associated with treatment-induced trismus, pain during mouth opening was reported by 100% of participants. After mouth opening, headache and restriction in cervical range of motion were reported in up to 15 participants (83%). Compensatory changes in posture and intraoral pain were reported by 14 participants (77%). Others, such as early muscle fatigue when chewing and referred pain to the ear, were also reported.

In terms of activities of daily living, in line with the most common clinical features, the most common response was difficulty while eating, with 100% of participants reporting this answer, followed by daily tooth brushing or oral care by 16 participants (88%) and difficulties when speaking (83%). Others such as sexual disturbances (50%), social isolation (39%) sleep disturbance due to jaw tension or nighttime pain (33%) were also reported.

### 3.3. Treatment Practices

In terms of treatment, 100% of respondents recommended the use of MT and TE as part of rehabilitation approaches for trismus management. As one participant explained: “*Depending on the mobility and condition of the tissues, we progressively advance from mobility work toward the development of strength, which could become a motivation for patients*”. In addition, 66% also recommended electrotherapy as a coadjuvant therapy, “*mainly for pain relief*”. 55% of participants also recommended the use of devices; the use of education in health and habits was reported as a recommendation by 50% of respondents, while specifically pain neuroscience education was recommended by six participants (33%).

Among manual therapy techniques, joint mobilization was suggested as useful by 100% of respondents. Other techniques such as myofascial therapy and intra and extraoral massage were recommended by nine (50%) and fourteen (77%). In addition, lymphatic drainage was added by 38% of participants.

Participants identified several exercise interventions as part of their rehabilitation approach. The most recommended was functional chewing exercises, with 100% of physiotherapists including this modality in their recommendations. As one expert stated: “*functional exercises are more engaging for patients and reflect daily life better than isolated jaw movements*”. This modality was followed by active range of motion exercises and counter-resistance exercises, included by 66% of respondents. Other exercise modalities such as static stretching and motor control exercises were recommended by 38% and 50% of participants, respectively. To implement functional exercises, different strategies were followed, such as initiating with mirror therapy or postural control, or progressing from mobility exercises towards the development of strength.

### 3.4. Barriers and Implementation of TE and MT

Participants identified several barriers to implementing TE and MT in clinical practice. Concerning manual therapy, a recurring theme was that edentulism or unstable processes may limit intraoral access and reduce patient tolerance to pressure. As it was stated: “*In patients with removable prostheses, it’s harder to apply guide movements or joint mobilizations without causing discomfort or losing biomechanical reference*.”

In addition, up to eight respondents (44%) noted that patients without dental occlusion may lack anatomical reference points for symmetrical assessment before and after treatment, thus reducing treatment precision. In addition, removable prostheses were also highlighted as a limitation, as it was explained: “*Intraoral joint mobilization in patients with removable prostheses must be applied with caution, since the resistance provided by patients is high, and force applied by physiotherapists may break the prosthesis*.”

Concerning TE, participants identified several limitations in the therapeutic exercise interventions currently implemented for patients with treatment-induced trismus. These limitations encompassed both structural and clinical aspects, with a criticism toward existing tools because of their lack of functionality and capacity of mimicking functional movements. Up to 13/18 answers were along these lines, highlighting “*The lack of tools to work on bite functionality makes it difficult to replicate the functional movement to be restored, limiting the capacity for soft tissue regeneration*.”

Other limitations to improving adherence to TE included socioeconomic factors, which can lead to the presence of psychological factors and, thus, limiting adherence. In that sense, the lack of health education was also pointed out as an important limitation to adherence, as it can be seen in this example: “*Social factors, such as socioeconomic level, are fundamental limitations. These are often people who feel desperate about their situation, with psychological factors such as depression that can limit adherence*.”

Qualitative responses highlighted various challenges in developing functional masticatory exercises. One of the most frequently mentioned barriers was the lack of dental stability (due to missing teeth or poorly adapted prostheses), which complicates the use of resistance-based exercises. One expert stated: “*Without stable posterior teeth, it’s very difficult to simulate functional chewing patterns. We often must adapt with more general mobility work*.”

### 3.5. Medical Devices in the Rehabilitation of Trismus

Up to 14/18 (77%) participants recommended the use of clinical devices in trismus rehabilitation. Among them, the Therabite device was the most used, with 12/14 (85%) of respondents. Another device used by more than 50% of experts was Restorabite, with 57% of physiotherapists recommending its use.

Despite its use, devices were criticized, especially because of their passive utility, without respecting joint biomechanics. In that sense, the lack of functionalitywas remarked upon by 94% of participants, and 100% of them thought that the lack of adaptability to the dental arch may become a limitation, as was often reported: “*Some devices only allow stretching, while those that do permit chewing exercises do not adapt to the dental arch, limiting the functionality of the movement and, therefore, its benefits*.”

In that sense, a common answer was directed toward the fact that chewing force is usually performed by molars and pre-molars, but these devices are usually developed to implement forces in central incisors. Indeed, some experts reported potential anatomical limitations: “*periodontal ligament may be highly forced with these kinds of devices*”.

## 4. Discussion

To the best of our knowledge, this is the first qualitative study to explore perspectives and clinical practices of physiotherapy experts in the management of treatment-induced trismus in people with HNC. Through a web-based survey, we obtained insights into the current state of rehabilitation modalities, strategies and perceptions about potential barriers, as well as into the use of medical devices. In that sense, a key finding from this study is the limited use of medical devices in clinical practice, despite the current state of research in which the use of these devices is supported by findings.

Although web-based qualitative and mixed-methods surveys have been used in other areas of physiotherapy such as musculoskeletal or neurological rehabilitation [21,22], the present study represents a novel contribution in the oncological and maxillofacial field. These types of surveys offer several benefits: they allow for the inclusion of geographically dispersed professionals, ensure participant anonymity, and facilitate the collection of both quantitative trends and rich, context-specific qualitative data. Such approaches are particularly useful in underexplored clinical areas where no standardized guidelines exist, enabling researchers to gather insights directly from clinical experts that can inform future practice and research priorities, in addition to exploring the perspectives of experts in how to apply evidence-based care [23].

Among our findings, we identified that trismus is not presented solely but accompanied by other clinical features. Among them, headache was one of the most reported, along with pain referred to the ear. In that sense, a recent review highlighted the major heterogeneity in clinical presentations of orofacial pain and headache in this population [24]. Nonetheless, it was found that neuropathic pain was frequently present, including symptoms such as burning, tingling, sharpness, electric shocks and paresthesia, among others, and probably because of radiotherapy. With a prevalence ranging between 13.1% [25] and 64.5% [26], the use of self-reported questionnaires such as the S-LANSS questionnaire may help with its diagnosis [27].

Findings from the present study are expected to show limited use of medical devices in the rehabilitation of trismus among physiotherapists, as preliminarily suggested by expert responses. This restricted usage could be attributed to the limited functionality of existing devices, such as Dynasplint [8], Therabite [28] or Restorabite [29], among others. Some experts have raised concerns that many of these tools rely solely on the use of the upper and lower central incisors to perform mouth-opening exercises, potentially placing excessive strain on the periodontal ligaments of these teeth, especially in vulnerable patients. Others have pointed out that this approach fails to mimic the natural biomechanics of chewing, which primarily involves the premolars and molars, which is in line with previous suggestions coming from literature reviews [30]. In that sense, our data revealed strong concerns regarding the functional relevance of the lack of adaptation to dental arches. This finding aligns with calls in the literature for more ergonomic, functionally relevant devices, which could limit the transferability of improvements to everyday activities such as eating and speaking which may lead to patient-centered device designs to increase adherence [17]. In addition, from a clinical perspective, our findings reinforce the importance of interdisciplinary collaboration. Optimal trismus rehabilitation may require coordinated care between physiotherapists, dentists (to address prosthetic stability and occlusion), speech and language therapists (to integrate swallowing training), and oncology teams. In terms of device development, physiotherapists emphasized the need for solutions that simulate functional chewing, distribute load to posterior teeth, and potentially incorporate feedback mechanisms. From an educational standpoint, incorporating training on trismus management, device use, and adherence strategies into physiotherapy curricula and continuing professional development could strengthen clinical practice.

Another important barrier to functional rehabilitation identified in this study is the absence of dental prostheses in some patients with HNC. Without these prostheses, the occlusal contacts necessary for functional mastication exercises are compromised, potentially delaying or even preventing the initiation of meaningful neuromuscular training of the masticatory system, with the related impact on nutritional habits in edentulous patients following oncologic treatment [31]. These limitations were also suggested in a previous systematic review in people with TMD [32], in which the absence of prostheses or the use of incorrectly adapted ones may limit the effectiveness of rehabilitation. Indeed, it was found that patients who received implant-supported dentures after mandibular reconstruction had greater masticatory efficiency, improved muscle activity, and a more symmetrical distribution of occlusal force, compared to removable prostheses. This is in line with our findings suggesting the importance of proper occlusion and prosthesis for the functional rehabilitation of trismus. From a physiotherapeutic perspective, this hinders the shift from passive mobilization to active and functional rehabilitation, particularly in protocols aiming to improve force production, range of motion, and coordination of the jaw musculature. Future clinical strategies should consider the dental status of each patient, and interdisciplinary collaboration with maxillofacial surgeons and dental specialists becomes essential to optimize timing and content of trismus rehabilitation.

In addition, our findings echo recent concerns in the literature that, despite the high prevalence of trismus and its functional consequences, clinical research has often failed to provide a strong theoretical or mechanistic rationale for rehabilitation approaches. As suggested recently, many interventions lack adequate justification and explanation of underlying mechanisms, limiting their translational value [30]. Notably, this may potentially explain why manual therapy, widely used in clinical practice (reported by 100% of participants in our study), remains underexplored in clinical trials. As previously published [33], myofascial therapy was recommended by up to 50% of experts. However, techniques such as manual mobilization, recommended by 100% of authors and included in clinical books [19] have not been studied in clinical studies, only in case series [34], hindering its potential benefit. Indeed, criticism was already given by a scoping review unravelling this lack of studies in the field of manual therapy [35]. Studies reporting benefits in preserving mouth opening have used therapeutic exercise, yet these trials did not include manual therapy modalities, leaving an important evidence gap [36,37].

### 4.1. Limitations

This study is not exempt from limitations. The exploratory nature of this study should be emphasized: the small sample size (*n* = 18), restriction to physiotherapists practicing in Spain, and reliance on self-reported experiences all limit the transferability of findings. Therefore, the results should be interpreted cautiously and considered as a starting point for further hypothesis generation rather than definitive conclusions. Secondly, while participants were selected in a step-by-step pre-defined algorithm, self-reported experience may be subject to bias, and no external validation of clinical background was performed. Third, the use of a web-based survey, although convenient and accessible, limited the possibility for follow-up or deeper probing that could be achieved in semi-structured interviews or focus groups, or even the in-depth analysis performed in a Delphi study [38].

Furthermore, intrinsic to the qualitative methodology, the interpretation of open-ended responses involves a level of subjectivity, despite the structured approach provided by thematic analysis. Although two researchers participated in the analysis and enhanced credibility, the absence of participant validation is another potential limitation.

Finally, due to the methodology and structure of the questionnaire, which was primarily designed to explore barriers, treatment modalities, and device limitations, relatively few facilitators or successful practices were reported by participants. This may reflect not only the content focus of the survey but also the tendency of clinicians to emphasize challenges when responding to open-ended questions.

### 4.2. Future Implications

From a clinical point of view, this study includes the need to promote access to and training in the use of medical devices, particularly in settings where resource constraints limit their availability. In addition, the need for retraining functionally mandibular chewing remains essential, and new devices may offer this possibility through innovative designs. To that end, new devices may be developed based on adaptation to the dental arch, simulating the chewing with molar and premolar dental pieces.

On another note, educational efforts to improve patient adherence and motivation may be taken into consideration, such as the inclusion of self-management strategies and better communication tools. This may lead to the transfer of rehabilitation to daily activities.

Concerning methodology, future studies should focus on developing intervention protocols combining MT with TE, with and without the use of devices, in the same way as including bite retraining. Moreover, not only effectiveness should be assessed, but also feasibility, acceptability and adherence, considering both clinicians’ and patients’ perspectives. In addition, future qualitative studies employing interviews or focus groups could provide a more balanced exploration of both barriers and facilitators.

Finally, including patient experiences into qualitative research would provide a more comprehensive understanding of rehabilitation challenges in this population.

## 5. Conclusions

This study provides physiotherapists’ perspectives on the management of treatment-induced trismus in HNC. Findings revealed that while MT and TE are core components of rehabilitation, their implementation is often challenged by patient-related factors, such as dental status, psychosocial barriers and other adherence factors. Their emphasis on functional chewing, stepwise progression from mobility to strength, and patient education suggests concrete ways to improve current protocols, both in research and clinical practice. In addition, perceived limitations in current medical devices also were reported, since these devices were described as lacking functional specificity, with concerns about poor adaptation to the dental arch and limited replication of masticatory patterns, potentially compromising clinical benefit. Clinically, rehabilitation should integrate manual therapy, exercise, and well-designed devices. Finally, strengthening physiotherapists’ training in trismus management and adherence strategies is equally important. Physiotherapists’ perspectives offer valuable exploratory insights that could inform the development of trismus rehabilitation protocols; however, confirmatory studies in broader and more diverse settings are needed before these approaches can be widely implemented.

## Figures and Tables

**Table 1:** Thematic Structure of the Final Questionnaire, Including Distribution of Closed- and Open-Ended Questions Across Five Sections.

Sociodemographic and Professional Information	Experience with Patients with HNC and Their Features	Assessment and Treatment Practices	Barriers in the Implementation of TE and MT	Medical Devices in the Rehabilitation of Trismus
Age	Years in the management of people with H&NC	Treatment modalities for trismus	Limitations to (a) MT and (b) TE because of dental prostheses	Use of medical devices
Gender	Patients treated per year with H&NC	MT modalities	Recommended strategies to develop functional chewing rehabilitation	Models used
Years of experience in patients with TMD	Main clinical features associated to trismus	TE modalities	Limitations for implementing and boosting adherence in TE	Limitations coming from these medical devices
-	ADL most affected by trismus	-	-	-
“How many years have you been practicing as a physiotherapist?”	“Do you use manual therapy in trismus rehabilitation? Please specify how.”	“Which exercise modalities do you prescribe (e.g., mobility, resistance, functional chewing)?”	“What are the main advantages or limitations you encounter with trismus devices?”	“In your experience, what factors hinder or facilitate patient adherence to rehabilitation?”

ADL: Activities of Daily Living; MT: Manual Therapy; TE: Therapeutic Exercise.

## Data Availability

The original contributions presented in this study are included in the article/Supplementary Material. Further inquiries can be directed to the corresponding author(s).

## Supplementary Materials

The following supporting information can be downloaded at: https://www.mdpi.com/article/10.3390/jcm14197092/s1, File S1: Full survey.

## Abbreviations

The following abbreviations are used in this manuscript:

MT	Manual Therapy
TE	Therapeutic Exercise
TMD	Temporomandibular Disorders
HNC	Head and Neck Cancer
