# Supplementary material for: Perceptions and Experiences of Physiotherapists Treating Trismus in Head and Neck Cancer Patients: Findings from a Spanish Web-Based Survey
Source: J Clin Med. 2025 Oct 8;14(19):7092. doi: 10.3390/jcm14197092 (PMC12525520; doi:10.3390/jcm14197092)
Supplement: Supplementary file 1 [file jcm-14-07092-s001.zip › jcm-3850846-supplementary.pdf]

Supplementary Material 1. Web-based survey

Encuesta Profesionales: Trismus en H&N Cáncer

Estás siendo invitado/a a participar en un estudio titulado:
"Percepciones, experiencias y uso de dispositivos en el tratamiento del trismus asociado al cáncer de cabeza y cuello por parte de 9sioterapeutas expertos".

Investigadora responsable: Ernesto Anarte Lazo, fisioterapeuta e investigador en Universidad UNIE.
Contacto: anartelazo.ernesto@gmail.com

Este estudio tiene como objetivo explorar las experiencias y percepciones de los @sioterapeutas con respecto al manejo del trismus asociado al tratamiento del cáncer de cabeza y cuello.

- Participación voluntaria, duración estimada: 8–10 minutos.
- Tus respuestas serán **anónimas y con9denciales**.
- Los datos se emplearán solo con @nes cientí@cos.

De acuerdo con lo establecido en el **Reglamento (UE) 2016/679 del Parlamento Europeo y del Consejo**, de 27 de abril de 2016 (**Reglamento General de Protección de Datos – RGPD**), y en la **Ley Orgánica 3/2018, de 5 de diciembre**, de Protección de Datos Personales y garantía de los derechos digitales (**LOPDGDD**), se garantiza que los datos recogidos en esta encuesta serán **tratados de forma totalmente anónima y con9dencial**, utilizados exclusivamente con @nes cientí@cos y académicos, y no serán cedidos a terceros bajo ninguna circunstancia. No se recopilarán datos personales identi@cativos, y en ningún caso se podrá asociar una respuesta con la identidad del/la participante.

~~* Indica que la pregunta es obligatoria~~

1. ¿Aceptas participar en este estudio bajo las condiciones descritas? * *Marca solo un óvalo.*

   - ⬭ Sí, consiento participar de forma voluntaria
   - ⬭ No deseo participar

2. ¿Cuál es tu género? * *Marca solo un óvalo.*

   - ⬭ Hombre
   - ⬭ Mujer
   - ⬭ No-binario
   - ⬭ Pre@ero no decirlo

3. ¿Cuál es tu edad?

____

4. ¿Cuántos años de experiencia clínica tiene en el manejo de trastornos temporomandibulares?

Marca solo un óvalo.

- Menos de un año
- 1-5 años
- 5-10 años
- Más de 10 años

5. ¿Cuántos años de experiencia clínica tiene en el manejo de pacientes con trismus asociad al tratamiento de cáncer de cabeza y cuello?

Marca solo un óvalo.

- Menos de un año
- 1-3 años
- 3-5 años
- Más de 5 años

6. ¿Cuántos años lleva investigando en el trismus asociado al cáncer de cabeza y cuello? * *Marca solo un óvalo.*

- Menos de un año
- 1-3 años
- 3-5 años
- Más de 5 años

7. ¿Qué volumen anual aproximado de pacientes de este tipo tratas, ya sea en clínica o en investigación?

Marca solo un óvalo.

- 0-20
- 20-50
- 50-100
- Más de 100

8. ¿Cuáles son las características clínicas asociadas a la limitación de la apertura con más influencia en la calidad de vida de estos pacientes? Indica 3 máximo.

Selecciona todos los que correspondan.

- Cefalea
- Dolor intrabucal
- Dolor a la apertura mandibular
- Crepitaciones o chasquidos mandibulares
- Cambios posturales compensatorios Limitación
- en la movilidad cervical
- Otro: __________

9. ¿Qué aspectos de la vida diaria o social crees que se ven más afectados por la limitación de la apertura mandibular en tus pacientes con trismus? Indica 3 máximo.

Selecciona todos los que correspondan.

- Di@cultad para reír, bostezar o mostrar emociones
- Di@cultad para comer
- Hablar o expresarse con claridad
- Cepillado dental o cuidado oral diario
- Afectación en la vida sexual o íntima
- Aislamiento social
- Otro: __________

Salta a la pregunta 10

Manejo del trismus asociado al tratamiento de cáncer de cabeza y cuello

10. ¿Qué tipo de intervenciones recomiendas para el manejo del trismus en este tipo de pacientes?

____

11. Dentro de la terapia manual, ¿qué técnicas recomiendas para el trismus? *

Selecciona todos los que correspondan.

- [ ] Movilización articular
- [ ] Terapia miofascial
- [ ] Masoterapia (intra y extrabucal)
- [ ] Otro: _______________________________

12. ¿Puedes explicar cómo afecta la presencia de prótesis dentales o la ausencia de piezas dentales a la forma de aplicar terapia manual en estos pacientes?

____

____

____

____

____

13. Dentro del ejercicio terapéutico, ¿qué intervenciones recomiendas para el manejo del trismus?

Selecciona todos los que correspondan.

- [ ] Estiramientos activos sostenidos
- [ ] Movilizaciones activas dinámicas para ganar rango de movimiento
- [ ] Ejercicios contra-resistencia
- [ ] Ejercicios funcionales para el trabajo masticatorio
- [ ] Otro:

14. ¿Qué estrategias recomiendas para el desarrollo de ejercicios funcionales como el trabajo masticatorio?

15. ¿Qué limitaciones consideras que tienen a día de hoy las intervenciones mediante ejercic terapéutico habitualmente implementadas en estos pacientes?

16. ¿Qué limitaciones consideras que existen para la adherencia a la prescripción de ejercicio terapéutico en estos pacientes?

17. ¿Puedes explicar cómo puede afectar la presencia de prótesis dentales o la ausencia de piezas dentales a la forma de implementar ejercicio terapéutico en estos pacientes?

Dispositivos médicos para la rehabilitación del trismus asociado al tratamiento de cáncer de cabeza y cuello

18. ¿Utilizas dispositivos médicos para el trabajo de rehabilitación del trismus? * *Marca solo un óvalo.*

- Sí
- No

19. En caso afirmativo, ¿qué dispositivo recomiendas?

Selecciona todos los que correspondan.

- [ ] Therabite Jaw Motion Rehabilitation System
- [ ] Dynasplint Trismus System
- [ ] EZBite device
- [ ] Restorabite device
- [ ] Otro: _______________

20. ¿Qué limitaciones consideras que presentan estos dispositivos para la mejora de la apertura mandibular?

____

____

____

____

____

21. ¿Crees que el hecho de que los dispositivos estudiados en investigación para el manejo del trismus no se adapten en su mayoría a la arcada dental puede suponer una limitación *Marca solo un óvalo.*

- ( ) Sí
- ( ) No

22. En caso afirmativo, ¿por qué?

____

23. Ciertos dispositivos desarrollados para permitir un trabajo masticatorio activo han sido diseñados para realizar esta fuerza con los incisivos centrales. ¿Crees que, al no realizarse el trabajo con la dentadura premolar y molar, puede convertirse en una limitación?

Marca solo un óvalo.

- Sí
- No

24. En caso afirmativo, ¿por qué?

Este contenido no ha sido creado ni aprobado por Google.

Google Formularios
